# The Effect of Automatic Gain Control Structure and Release Time on Cochlear Implant Speech Intelligibility

**DOI:** 10.1371/journal.pone.0082263

**Published:** 2013-11-28

**Authors:** Phyu P. Khing, Brett A. Swanson, Eliathamby Ambikairajah

**Affiliations:** 1 School of Electrical Engineering and Telecommunications, The University of New South Wales, Sydney, Australia; 2 Cochlear Limited, Sydney, Australia; McGill University, Canada

## Abstract

Nucleus cochlear implant systems incorporate a fast-acting front-end automatic gain control (AGC), sometimes called a compression limiter. The objective of the present study was to determine the effect of replacing the front-end compression limiter with a newly proposed envelope profile limiter. A secondary objective was to investigate the effect of AGC speed on cochlear implant speech intelligibility. The envelope profile limiter was located after the filter bank and reduced the gain when the largest of the filter bank envelopes exceeded the compression threshold. The compression threshold was set equal to the saturation level of the loudness growth function (i.e. the envelope level that mapped to the maximum comfortable current level), ensuring that no envelope clipping occurred. To preserve the spectral profile, the same gain was applied to all channels. Experiment 1 compared sentence recognition with the front-end limiter and with the envelope profile limiter, each with two release times (75 and 625 ms). Six implant recipients were tested in quiet and in four-talker babble noise, at a high presentation level of 89 dB SPL. Overall, release time had a larger effect than the AGC type. With both AGC types, speech intelligibility was lower for the 75 ms release time than for the 625 ms release time. With the shorter release time, the envelope profile limiter provided higher group mean scores than the front-end limiter in quiet, but there was no significant difference in noise. Experiment 2 measured sentence recognition in noise as a function of presentation level, from 55 to 89 dB SPL. The envelope profile limiter with 625 ms release time yielded better scores than the front-end limiter with 75 ms release time. A take-home study showed no clear pattern of preferences. It is concluded that the envelope profile limiter is a feasible alternative to a front-end compression limiter.

## Introduction

The electrical dynamic range of a cochlear implant recipient, between the threshold current (T-level) and the maximum comfortable current (C-level), is typically 10 – 20 dB [1]. This is much less than the range of sound levels encountered in the environment. The dynamic range of speech for a single talker is 40 – 50 dB [2], [3]. The overall level varies by about 30 dB across talkers, from casual conversation to shouting [4]. Thus a cochlear implant system requires some form of compression or automatic gain control (AGC).

**Figure 1:**
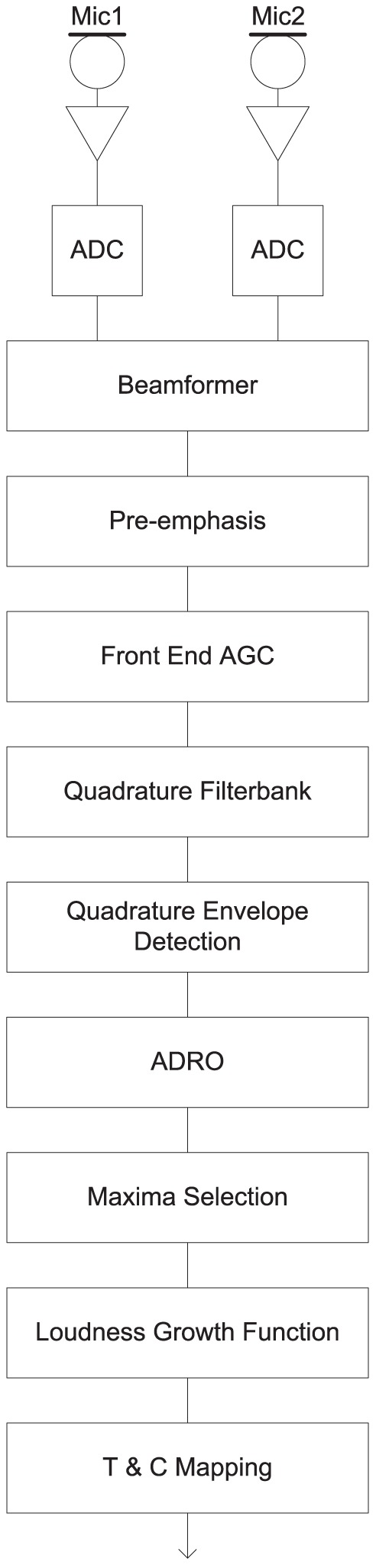
Nucleus cochlear implant system signal path. Mic = Microphone, ADC = Analog-to-Digital Converter, AGC = Automatic Gain Control, ADRO = Adaptive Dynamic Range Optimisation, T & C = Threshold and Comfort level.

**Figure 2:**
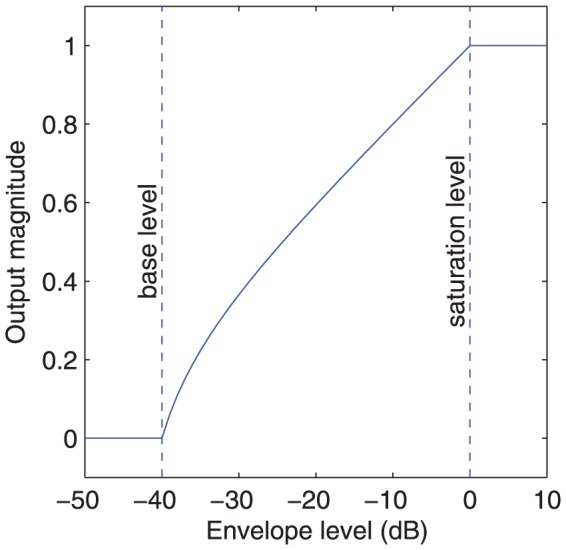
Loudness Growth Function (LGF). The LGF maps filter envelope levels to output magnitudes in the range 0 to 1. The output magnitude is the proportion of the electrical dynamic range on each channel. The saturation level is the envelope level that produces a magnitude of 1, which then yields current at C-level. The base level is the envelope level that produces a magnitude of 0, which then yields current at T-level.

The signal path used for the Advanced Combinational Encoder (ACE) sound coding strategy in Nucleus cochlear implant systems (Cochlear Limited, Sydney, Australia) is shown in Figure 1 [5], [6]. Signals from dual microphones are sampled by two 16-bit analog-to-digital converters (ADCs) at 15.7 kHz and combined in the beamformer to provide either fixed or adaptive [7] directionality. The pre-emphasis filter is designed to flatten the long-term average speech spectrum. The front-end AGC includes a slow-acting Automatic Sensitivity Control (ASC) [8], followed by a fast-acting compression limiter (i.e. an AGC with infinite compression ratio). The filter bank contains a pair of quadrature band-pass filters for each of up to 22 electrodes, followed by quadrature envelope detection [6]. Adaptive Dynamic Range Optimization (ADRO) [9]–[11] estimates the peak level, average level, and background noise level of each filter bank output envelope over intervals of several seconds. It slowly varies the gain on each frequency band independently to maintain a comfortable level. The Maxima Selection block examines the envelopes in each analysis period and selects those with the largest amplitude (typically 8 – 12 of the 22 channels) for stimulation. The Loudness Growth Function (LGF) is an instantaneous non-linear compressive function that defines the mapping from envelope levels to currents (Figure 2). It compresses a (typically) 40 dB dynamic range into the available range of stimulation current. The shape of the LGF is intended to make the cochlear implant recipient's loudness perception match that of a normal hearing person for changes in sound intensity. To avoid excessive loudness, the stimulation current is not allowed to exceed C-level. The LGF saturation level is the envelope level that produces current at C-level.

The signal path is calibrated with the intent that speech at 65 dB SPL results in envelope levels just reaching the LGF saturation level, so that stimulation currents just reach C-level. If there was no AGC, then speech at higher presentation levels would produce envelope levels exceeding the LGF saturation level, and the envelope waveforms would be clipped. The purpose of the compression limiter is to avoid this envelope distortion, and therefore the compression threshold should correspond to the LGF saturation level. However, this cannot be achieved consistently, because the envelope levels depend on the crest factor (i.e. the peak-to-rms ratio) and bandwidth of the audio signal.

The present study investigated the feasibility of replacing the front-end AGC with a multichannel AGC. Bringing together all the gain control elements at a single point in the signal path (after the filter bank) may enable simplification and optimization. ASC and ADRO, which both have time constants of several seconds, could be combined. It may also allow better integration of new features such as SNR-based noise reduction [12] and dual-microphone spatial noise reduction [13], which also act on the filter bank outputs. A further potential benefit is that monitoring signal levels at the input to the LGF allows the compression threshold to be set precisely equal to the LGF saturation level, so that envelope clipping can be eliminated. It was hypothesized that this would provide better speech intelligibility.

A secondary goal of the present study was to investigate the effect of AGC speed on cochlear implant speech intelligibility. AGC speed was specified by the release time, the time taken for the gain to recover to within 2 dB of its final value after a decrement in the input level from 80 to 55 dB SPL (IEC 60118-2). There is no clear consensus regarding the best AGC speed in acoustic hearing aids [14]–[17]. Furthermore, acoustic hearing aid results are not necessarily applicable to cochlear implants.

Stone and Moore [18]–[20] studied normal-hearing subjects listening to noise vocoder simulations of cochlear implant processing, and found that fast AGC degraded intelligibility in the presence of a competing talker. The dominant cause was cross-modulation, whereby fluctuations in the level of one talker produced correlated fluctuations in the level of the other, making it harder to segregate the two talkers. McDermott et al. [21] showed benefits of a fast AGC system for cochlear implant recipients in quiet, due to increased audibility of low level speech components. However, there was a suggestion that intelligibility in noise was degraded, with 7 out of 10 recipients having worse performance with compression.

Hearing aids often use multichannel AGCs because the amount of hearing loss, and the dynamic range of residual hearing, varies with frequency. However, if each channel operates independently, with fast time constants, then amplitude differences across frequencies will be reduced, degrading the spectral cues used in recognising speech. Plomp [22] tested both normal hearing and hearing impaired listeners with multichannel fast AGC. Sentence in noise scores reduced monotonically as the number of channels increased from 1 to 16, and as the compression ratio increased. Stone and Moore [19], using vocoder simulations, observed worse intelligibility for an 11-channel fast AGC compared to a front-end AGC. In a subsequent study [23], they found that intelligibility decreased as the number and speed of the compression channels increased.

Given these results, it would be expected that independent fast AGC on 22 channels would give very poor performance. A solution is to cross-couple the channels, so that the gains are related in some way [24]. The multichannel AGC in the present study took this approach.

The disadvantages of a fast AGC can be alleviated by a dual-loop AGC [25], [26]. It consists of a slow AGC, to handle long term level variations, together with a fast AGC to handle intense transients. Stöbich et al. [27] found that cochlear implant speech intelligibility was better with a dual-loop AGC than with a slow AGC when speech was preceded by a brief intense sound. Boyle et al. [28] found that a dual-loop AGC gave better cochlear implant speech intelligibility than a fast AGC in sentence tests with both fixed and roving presentation levels.

In early Nucleus processors (such as Spectra, ESPrit, and Freedom), ASC used a noise floor estimator to slowly vary the gain [8]. A brief intense sound had negligible effect on the ASC gain, but activated the fast compression limiter that followed; the overall behaviour was comparable to a dual-loop AGC. In the CP810 processor (used in the present study), the front-end AGC is similar in design to the “DUAL-HI” dual-loop AGC described by Stone et al. [26]. The term “ASC” was retained to refer to the slow stage of the dual-loop AGC, which has the same time constants as the earlier ASC implementation. Both ASC implementations have been shown to substantially improve speech intelligibility in noise [29], [30].

Similarly, a combination of slow and fast time constants in a multichannel signal path would be expected to be beneficial. ADRO satisfies the need for slow multichannel AGC, and has been studied extensively [9]–[11]; hence the present study focussed on the fast multichannel AGC. A dual-loop system provides benefit because the fast AGC is activated relatively infrequently; however the fast AGC will operate whenever there is a sudden increase in the speech level, so it is still worthwhile understanding its effect.

## Methods

This study investigated two AGC structures (a traditional front-end compression limiter and a novel multichannel compression limiter) and two release times (75 and 625 ms). There were three experiments. Experiment 1 was a two-factor design, measuring sentence recognition in quiet and in noise, at a high presentation level chosen to maximize the compressive activity of each AGC system. Experiment 2 measured sentence recognition in noise as a function of presentation level, for two of the AGCs from Experiment 1. As the goal was to investigate the effect of fast AGC, the usual slow gain control blocks in the Nucleus signal path (ASC and ADRO) were disabled in Experiment 1 and 2. Experiment 3 was a take-home study of the multichannel compression limiter.

### Subjects

Seven Nucleus cochlear implant users participated in this study. Their demographic information and stimulation parameters are listed in Table 1. All subjects were experienced with their implant, were regular users of the ACE coding strategy on the CP810 processor, and were familiar with speech tests from previous studies.

**Table 1:** Demographic details and stimulation parameters for the seven CI subjects.

ID	Age (yrs)	Implant use (yrs)	Implant Type	Number of Channels	Number of Maxima	Stimulation Rate (Hz)
S1	75.6	5.4	CI24RE	20	10	1800
S2	86.0	12.1	CI24M	22	12	720
S3	74.3	2.8	CI24RE	22	8	900
S4	70.7	3.8	CI24RE	22	8	900
S5	49.4	6.8	CI24R	22	9	1200
S6	64.4	5.6	CI24M	22	12	900
S7	41.3	2.8	CI512	22	8	900
**Mean**	**66.0**	**5.6**				

### Ethics statement

Approval for the study was obtained from the Ethics Review Committee of Royal Prince Alfred Hospital, Sydney, and each participant provided written informed consent.

### Signal Processing

**Figure 3:**
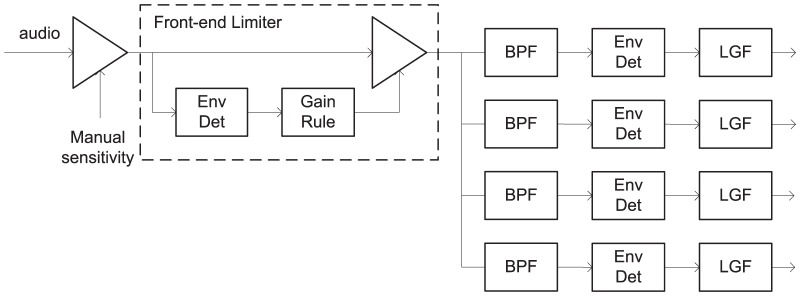
Block diagram of signal path with front-end limiter. BPF = Band-pass filter, Env Det = Envelope detector, LGF = Loudness Growth Function. For brevity, only four channels are shown, but recipients used 20 to 22 channels.

The front-end compression limiter was the same as that used in the Nucleus Freedom processor [5]. Figure 3 is a block diagram of the relevant section of the signal path with the front-end limiter. It had unity gain up to the compression threshold, and infinite compression beyond. For calibration purposes, the compression threshold for a 1 kHz pure tone was 73 dB SPL. Since the crest factor of speech is about 8 dB higher than that of a sinusoid, peaks of speech presented at 65 dB SPL sometimes reached the compression threshold. The attack time was 5 ms. The front-end limiter incorporated an overshoot limiter that constrained the maximum overshoot during an attack to 3 dB above the compression threshold. Two release times were tested in Experiment 1: a short release time of 75 ms (as in the Freedom processor), and a longer release time of 625 ms.

**Figure 4:**
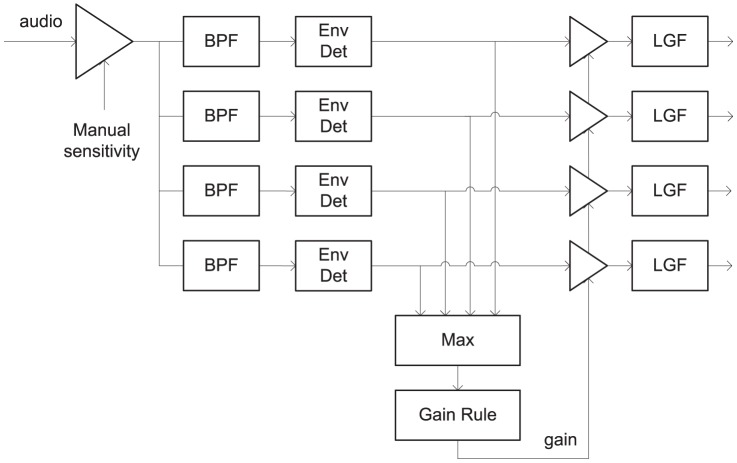
Block diagram of signal path with envelope profile limiter. BPF = Band-pass filter, LGF = Loudness Growth Function. Env Det = Envelope detector. For brevity, only four channels are shown, but recipients used 20 to 22 channels.

Figure 4 is a block diagram of the relevant section of the signal path with the proposed multichannel AGC. The Max block produced the instantaneous maximum value, across channels, of the set of envelopes, and the gain was based upon whichever channel had the largest amplitude. The gain was unity up to the compression threshold, and infinite compression was applied beyond. The resulting gain was then applied to all channels. For calibration purposes, the compression threshold for a 1 kHz pure tone was 59 dB SPL, so that when speech was presented at 65 dB SPL, the envelopes on some channels sometimes reached the compression threshold. A zero attack time was used, because rapid gain changes after the filter bank cannot produce any undesirable spectral smearing. The rise time of the envelopes was thus determined by the filter bank, and no overshoot could occur. The release time was either 75 ms or 625 ms.

With all channels having equal gain, at first glance it may appear that the multichannel AGC would have behaviour identical to that of the front-end limiter. The difference is that levels were measured after the filter bank, where they directly control the stimulation current, and the compression threshold was set equal to the saturation level of the LGF. Thus no envelope could exceed the LGF saturation level. Because it eliminates envelope clipping, and preserves the spectral profile, this multichannel AGC is referred to as an envelope profile limiter.

**Figure 5:**
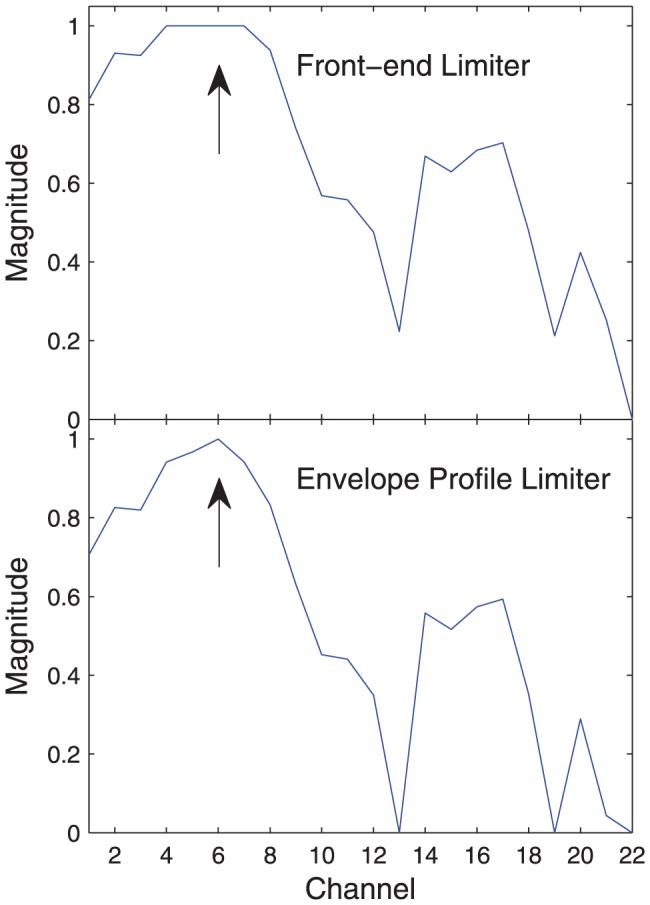
Envelope clipping – spectral effect. The 22-channel Loudness Growth Function (LGF) output at one time instant during the vowel in the word “locked”. The envelope profile limiter (bottom panel) preserves the spectral profile, with a peak on channel 6, indicated by the arrow. The front-end limiter (top panel) results in four envelopes (channels 4, 5, 6, and 7) hitting the saturation level, flattening the spectral peak.

**Figure 6:**
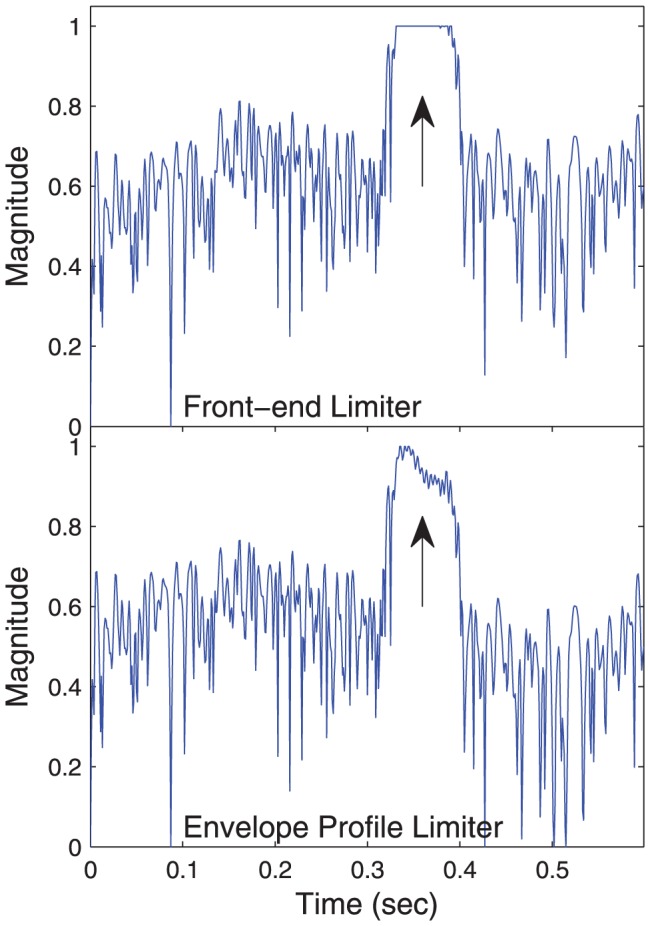
Envelope clipping – temporal effect. Waveform at output of Loudness Growth Function (LGF) on channel 4, centred at 625 Hz, for the word “locked” in noise. The envelope profile limiter (bottom panel) preserves the vowel modulation, indicated by the arrow. The front-end limiter (top panel) results in the envelope being clipped.

The behaviour of the two AGCs is compared in Figures 5 and 6. At high presentation levels, the front-end limiter allows some envelope clipping. This has three detrimental effects. Firstly, it distorts the spectral profile. As shown in Figure 5, it flattens spectral peaks, making it harder to determine formant frequencies, and potentially degrading vowel perception. Secondly, examining the temporal waveform in Figure 6, the amplitude modulation is lost. For a vowel, this modulation occurs at the fundamental frequency, and is the primary cue to voice pitch. Thirdly, at positive SNRs, envelope clipping reduces the amplitude of the signal peaks relative to the background noise, thus reducing the SNR. The envelope profile limiter avoids these drawbacks, and it was hypothesized that it would provide better speech intelligibility.

The envelope profile limiter is also computationally efficient. Because it acts on the envelopes, it can run at the envelope sampling rate, which is generally related to the stimulation rate (e.g. 1000 Hz) [6]; in contrast, the front-end limiter must run at the audio sampling rate. Furthermore, the operation of finding the largest envelope (Max block in Figure 4) is already required in the ACE sound coding strategy (Maxima Selection block in Figure 1).

### Test Set-up

For experiments 1 and 2, the signal path was implemented on a real-time research system, based on the Mathworks Simulink-xPC platform. Two omnidirectional microphones mounted in the behind-the-ear housing of a CP810 sound processor were wired to an external pre-amplifier, then applied to a high quality ADC. Stimulation commands were streamed to the subject's implant by a custom stimulation generator unit with an RF coil driver.

Two test set-ups were used. In the first set-up (referred to as the loudspeaker set-up), the audio was presented from a single loudspeaker one metre in front of the subject. The sound pressure level was restricted to 80 dB to avoid loudspeaker distortion. To achieve effective presentation levels above 80 dB SPL, the manual sensitivity control (see Figure 3 and Figure 4) was increased. The highest presentation level of sentences in this study was 89 dB SPL, a combination of 80 dB SPL acoustic level from the loudspeaker, and 9 dB additional gain from the manual sensitivity adjustment. All recipients used the Standard directionality [31].

The second set-up (referred to as the direct connect set-up) bypassed the loudspeaker and microphones, and presented the audio signal directly to the ADC of the real-time processing platform. A pre-emphasis filter was used to match the frequency response of the Standard directionality. This had two advantages: the audio could be presented at high levels (again, up to 89 dB SPL) without distortion, and there was no possibility of the recipient using any residual acoustic hearing in their contralateral ear. The drawback was that the subjects could not hear their own voices.

### Study Design

Experiments 1 and 2 used a repeated measure, single-subject design in which each subject served as their own control. The test order was counterbalanced between subjects. Subjects were not informed as to which AGC was being tested.

Listening tests were carried out in a sound-treated room. The sentence materials of the Australian Sentence Test In Noise (AuSTIN) were used [32]. Sixteen sentences were presented for each test condition. Each sentence was scored on the number of morphemes correctly repeated. For example, the sentence “She is do/ing her home/work” contains seven morphemes. Four-talker babble was used for the speech in noise test. Both the sentence presentation level and the SNR were fixed for each list of sentences. The background noise began one second before each sentence and finished one second after each sentence.

#### Experiment 1: High presentation level

Experiment 1 compared the front-end limiter and the envelope profile limiter, each with two release times (75 and 625 ms). Abbreviations for the four AGC configurations are listed in **Table 2**. To generate the most compressive activity from each AGC system and maximize any performance differences, a high presentation level of 89 dB SPL was used. All subjects used the direct connect set-up.

**Table 2:** AGC configurations tested.

AGC Type	Release Time (ms)	
	75	625
Front-end limiter	FE75	FE625
Envelope profile limiter	EP75	EP625

Sentences were presented in two conditions: in quiet, and in four-talker babble at 10 dB SNR. The speech-in-quiet condition reveals the effects of envelope distortions, in particular envelope clipping, as well as reduced amplitude modulation depth. The speech-in-noise condition is also subject to envelope distortion, and in addition, a fast AGC can worsen the effective SNR by amplifying the noise during speech pauses. The research system allowed the signal level at the input to the LGF to be monitored, so that the amount of envelope clipping could be measured.

#### Experiment 2: Performance-intensity function

The high presentation level used in experiment 1 was not representative of everyday listening conditions. The objective of experiment 2 was to measure performance over a wide range of presentation levels, i.e. to obtain performance-intensity functions. Because of the limited availability of the subjects' time, only two AGC configurations were tested: FE75, the Freedom processor baseline condition, which gave the lowest scores in experiment 1; and EP625, which gave the highest scores in experiment 1. The two AGCs were evaluated at presentation levels from 55 to 89 dB SPL, in four-talker babble, at two SNRs: 10 and 20 dB. The 20 dB SNR condition was used, instead of the speech-in-quiet condition of experiment 1, to avoid ceiling effects.

All subjects were initially tested with the loudspeaker set-up. Subject S1 obtained surprisingly good scores at the higher presentation levels, apparently assisted by his residual contralateral hearing (despite that ear being plugged), and therefore he was retested using the direct connect set-up.

#### Experiment 3: Take-home study

To complement the sound-room testing, a take-home study was conducted to investigate the performance of the envelope profile limiter in real-life listening conditions. Subjects were provided with a CP810 sound processor with research firmware that supported the envelope profile limiter. One program slot contained a standard program, using the standard front-end dual-loop AGC system (comprising ASC and the fast front-end compression limiter, i.e. FE75) and ADRO, as shown in Figure 1. A second program slot had the envelope profile limiter (with 625 ms release time), followed by ADRO. ADRO was kept at the same point in both signal paths (immediately before the LGF) because the goal was to study the effect of replacing the front-end AGC with the envelope profile limiter. All non-AGC program parameters were identical.

Each subject had the processor for at least two weeks. Subjects could switch between programs using a button on their processor or a remote control, and were encouraged to try both programs in many different listening scenarios. The subjects completed the HEARing Cooperative Research Centre in-house Comparative Performance Questionnaire (CPQ) [31], which asks them to rate the helpfulness of each program in a variety of listening situations. Each item in the questionnaire is rated on a five-point response scale, ranging from 1 (not helpful) to 5 (extremely helpful). The subject could select ‘Not Applicable’ if they did not experience that listening condition. In subsequent analysis, the benefit score for each question was defined as the rating for the envelope profile limiter program minus the rating for the standard program, giving a score in the range −4 to +4. In addition to the helpfulness ratings, the subjects were also asked to nominate their overall preferred program, and to rate its sound quality, in both quiet and noisy conditions. The rating was on a four-point scale: the preferred program was (1) very similar to, (2) slightly better than, (3) moderately better, or (4) much better than the other program.

### Statistical analysis

Sentence scores do not follow a normal distribution, making t-tests inappropriate. Instead, the resampling or bootstrap method [33], [34] was applied, using the “bootstrp” function from the MATLAB Statistics toolbox. This method makes no assumptions about the distribution of scores. Consider determining whether there was a significant difference in performance between two conditions for a particular subject in experiment 1. The subject provided a set of 16 sentence scores for each condition. The null hypothesis was that the two conditions yielded identical performance. If the null hypothesis was true, then the pooled set of 32 sentence scores reflected this common performance level. The objective was to estimate the likelihood that the observed difference in scores was due to random variation. A random sample of 16 scores was chosen, with replacement, from the set of 32, and the mean score was calculated. Then a second random sample of 16 was taken, and the difference between the two mean scores calculated. This process was repeated a large number of times, to give a vector of simulated score differences. An entry in this vector was classified as an extreme value if it was greater than or equal to the actual difference between the two mean scores. Finally, the p-value was calculated as [34]: p = (x+1)/(N+1) where x was the number of extreme values, and N was the number of replications, which was 9999 (thus the most significant possible p-value was 0.0001).

A group mean score for each condition was obtained by averaging scores across subjects. The effects of AGC type and release time were quantified by subtracting the appropriate group means. The p-values indicating whether these differences were significant were obtained by applying the same averaging operations to the vectors of simulated score differences, and then counting the corresponding extreme values.

## Results

### Experiment 1: High presentation level

**Figure 7:**
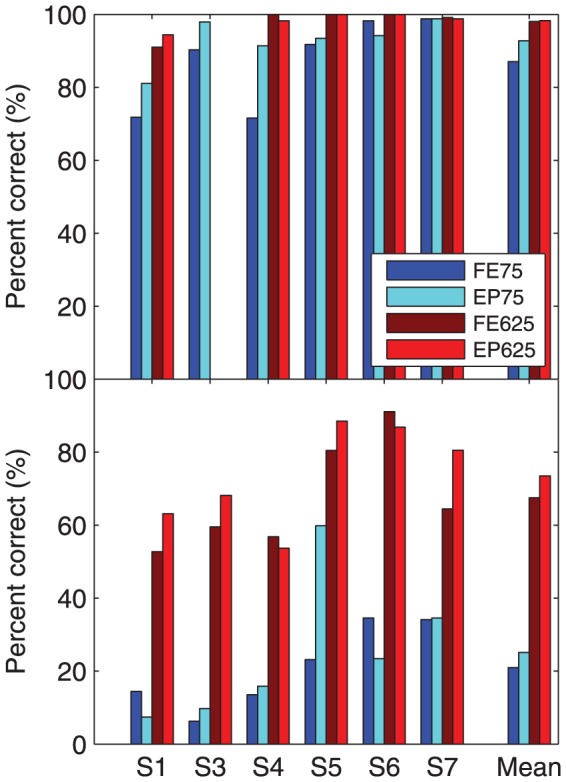
Results of experiment 1. Individual and group mean percent correct scores for sentences presented at 89(top panel) and in four-talker babble noise at 10 dB SNR (bottom panel), for four AGC configurations (abbreviations given in Table 2).

Six cochlear implant subjects participated in experiment 1. Figure 7 shows the mean percent correct scores of the individual subjects and the group means for sentences presented in quiet and in noise for the four AGC configurations. **Table 3** shows the differences between subject scores, across conditions, where the differences were statistically significant, both for individual subjects, and for the group.

**Table 3:** Pairwise comparisons of scores in experiment 1 that showed a significant difference. Column 5 is the difference between the observed scores.

Condition	Effect	Comparison	Subject	Percentage points difference	p-value
In quiet	Effect of release time	FE625 - FE75	S1	19	0.0328
		FE625 - FE75	S4	28	0.0068
		FE625 - FE75	S5	8	0.0351
		FE625 - FE75	Group	12	0.0001
		EP625 - EP75	S1	13	0.0491
		EP625 - EP75	S5	7	0.0462
		EP625 - EP75	Group	7	0.0001
	Effect of AGC type	EP75 - FE75	S3	8	0.0481
		EP75 - FE75	S4	20	0.0405
		EP75 - FE75	Group	6	0.0327
In noise	Effect of release time	FE625 - FE75	S1	38	0.0009
		FE625 - FE75	S3	53	0.0002
		FE625 - FE75	S4	43	0.0010
		FE625 - FE75	S5	57	0.0001
		FE625 - FE75	S6	57	0.0001
		FE625 - FE75	S7	30	0.0231
		FE625 - FE75	Group	46	0.0001
		EP625 - EP75	S1	56	0.0001
		EP625 - EP75	S3	58	0.0001
		EP625 - EP75	S4	38	0.0043
		EP625 - EP75	S5	29	0.0063
		EP625 - EP75	S6	63	0.0001
		EP625 - EP75	S7	46	0.0005
		EP625 - EP75	Group	48	0.0001
	Effect of AGC type	EP75 - FE75	S5	37	0.0038

Column 6 indicates whether the observed difference was statistically significant.

#### Without noise

Scores in quiet exhibited a ceiling effect, especially for the 625 ms release time. S3 did not undertake the 625 ms release time condition due to time constraints and because his scores were likely to have been near ceiling; the mean scores for the 625 ms condition shown in the upper panel of Figure 7 are for the remaining subjects.

The release time of 625 ms provided equal or better scores in quiet than the release time of 75 ms for all subjects, across both AGC types. Referring to **Table 3**, the improvement for FE625 over FE75 was significant for S1, S4, and S5, and the group mean showed a highly significant improvement of 12 percentage points (p = 0.0001). The improvement for EP625 over EP75 was significant for S1 and S5, and the group mean showed a highly significant improvement of 7 percentage points (p = 0.0001).

Regarding the effect of AGC type, EP75 provided significantly better scores in quiet than FE75 for subjects S3 and S4 (**Table 3**), and the group mean showed a significant improvement of 6 percentage points (p = 0.03).

#### With noise (at 10 dB SNR)

As expected, the addition of noise caused a substantial drop in performance. Release time had a pronounced effect, with 75 ms giving significantly worse scores than 625 ms for every subject, for both AGC types (**Table 3**). The group mean for FE75 was 46 percentage points lower than for FE625 (p = 0.0001), and the group mean for EP75 was 48 percentage points lower than for EP625 (p = 0.0001).

Regarding the effect of AGC type, S5 obtained a 37 percentage point improvement with EP75 over FE75 (**Table 3**). However, there was no significant difference between the group mean scores for the envelope profile limiter and the front-end limiter at either release time.

In summary, experiment 1 showed that a longer release time gave better speech intelligibility in both quiet and noise. Compared to the front-end limiter, the envelope profile limiter gave equivalent performance in noise, and showed a small benefit in quiet.

### Experiment 2: Performance-intensity function

**Figure 8:**
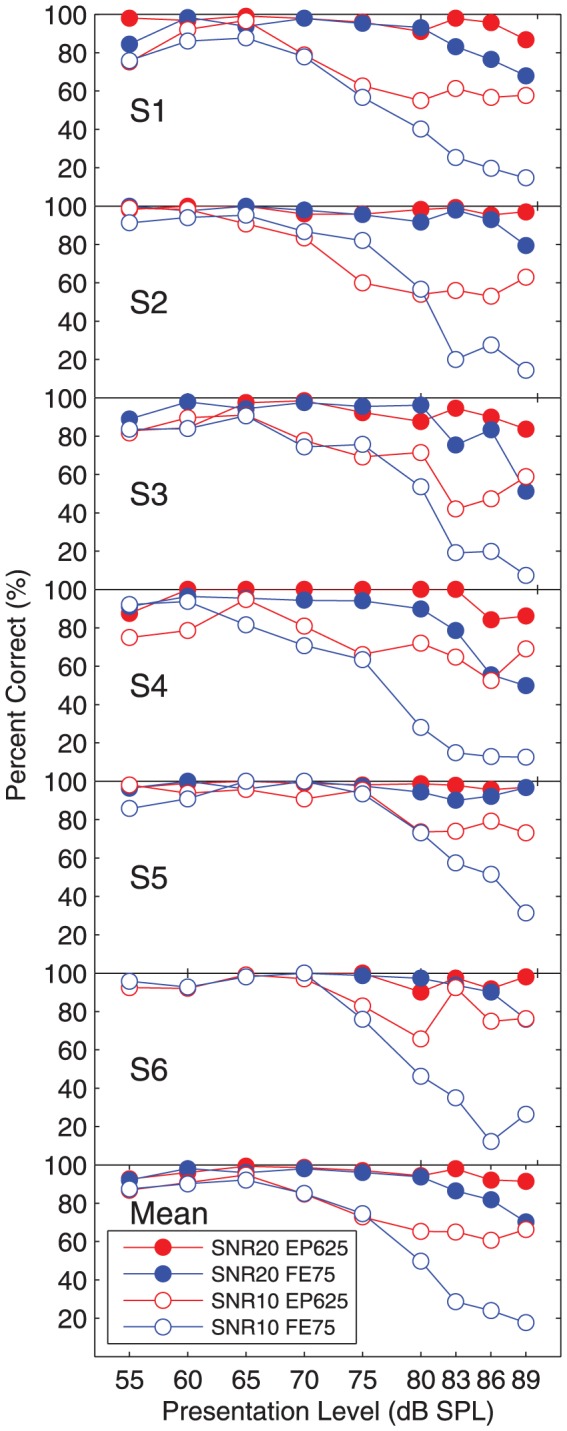
Results of experiment 2. Percent correct scores for sentences presented in four-talker babble noise for each subject (top six panels) and the group mean (bottom panel), for two AGC configurations (abbreviations given in Table 2). Filled symbols show results with an SNR of 20 dB, open symbols an SNR of 10 dB.

Six subjects participated in experiment 2, five of whom had taken part in experiment 1. Figure 8 shows the percent correct scores of the individual subjects and the group mean scores. **Table 4** shows the differences between subject scores, across conditions, where the differences were statistically significant, both for individual subjects, and for the group.

**Table 4:** Pairwise comparisons of scores in experiment 2 that showed a significant difference. All comparisons are EP625 – FE75.

SNR	Subject	Percentage points difference	p-value
20	S1	18	0.0001
20	S2	7	0.0116
20	S3	19	0.0013
20	S4	29	0.0008
20	S6	9	0.0177
20	Group	14	0.0001
10	S1	33	0.0001
10	S2	27	0.0002
10	S3	30	0.0001
10	S4	47	0.0001
10	S5	22	0.0008
10	S6	47	0.0001
10	Group	34	0.0001

Column 3 is the difference between the observed scores. Column 4 indicates whether the observed difference was statistically significant.

#### Performance-intensity function at 20 dB SNR

For presentation levels below the compression threshold of 65 dB SPL, both AGCs acted as unity gain amplifiers, and as expected, yielded almost identical group mean scores. Group mean scores for both AGCs were close to ceiling for presentation levels from 55 to 80 dB SPL. When the level was increased from 80 to 89 dB SPL, group mean scores with FE75 dropped by 24 percentage points, but scores with EP625 were maintained at a high level. Statistical analysis was applied to scores averaged across presentation levels 83 to 89 dB SPL (**Table 4**). Scores were lower with FE75 than with EP625 for all subjects (the difference being significant for all except S5), and the group mean score for FE75 was 14 percentage points lower than for EP625 (p = 0.0001).

#### Performance-intensity function at 10 dB SNR

As expected, scores at 10 dB SNR were substantially lower than scores at 20 dB SNR. Group mean scores were almost identical for the two AGCs for presentation levels from 55 to 75 dB SPL. The highest scores with both AGCs were obtained at 65 dB SPL. As the presentation level was increased to 89 dB SPL, the group mean scores with FE75 dropped by approximately 70 percentage points, but scores with EP625 dropped to a lesser extent, approximately 30 percentage points. Statistical analysis was applied to scores averaged across presentation levels 80 to 89 dB SPL (**Table 4**). Scores were significantly lower with FE75 than with EP625 for all subjects, and the group mean score for FE75 was 34 percentage points lower than with EP625 (p = 0.0001).

In summary, experiment 2 showed that as presentation level rose, intelligibility deteriorated with FE75, but was more robust with EP625.

### Experiment 3: Take-home study

Five cochlear implant recipients, S1, S3, S4, S5 and S7, participated in the take-home study. S1, S3 and S7 use contralateral hearing aids. S5 uses bilateral implants but only one processor had the envelope profile limiter program. From the questionnaire, seven questions concerning conversation in quiet, and six questions concerning conversation in noisy conditions were selected for analysis. **Table 5** shows the mean benefit score and the overall preferred program in quiet and noisy backgrounds. According to a t-test, the mean benefit score was not significantly different from zero. There was no clear pattern of preferences. Despite S7 showing a net benefit for the envelope profile limiter program in quiet, he still preferred the standard program. Some subjects reported anecdotally that background noise was more objectionable with the envelope profile limiter program. With the envelope profile limiter program, some subjects noticed sound drop-outs following impulsive sounds, for example door slams.

**Table 5:** Mean benefit scores and preferred program for experiment 3 – take home study – in quiet and noisy backgrounds.

ID	Quiet		Noisy	
	Preferred program	Mean benefit score	Preferred program	Mean benefit score
**S1**	Standard	−1	None	0.25
**S3**	None	0	EP	0
**S4**	-	-	-	−1
**S5**	EP	0.3	Standard	−1
**S7**	Standard	1.6	Standard	−0.8
**Mean**		0.2		−0.5

A positive mean benefit score means the subject rated the envelope profile limiter program (EP) as having greater benefit than the standard program. S4 did not answer some questions, as indicated by dashes.

## General Discussion and Conclusions

A front-end compression limiter prevents the amplitude of the audio signal from exceeding the compression threshold. However, if the signal path is calibrated for typical speech signals, then occasional envelope clipping can occur when the audio signal has narrow bandwidth or low crest factor. The proposed envelope profile limiter eliminated envelope clipping by monitoring the maximum envelope level (rather than the front-end level) and setting the envelope compression threshold to be equal to the saturation level of the LGF. It preserved the spectral profile by applying the same gain to all channels. The primary conclusion of this study is that the envelope profile limiter is a feasible alternative to a front-end compression limiter in a cochlear implant system.

**Figure 9:**
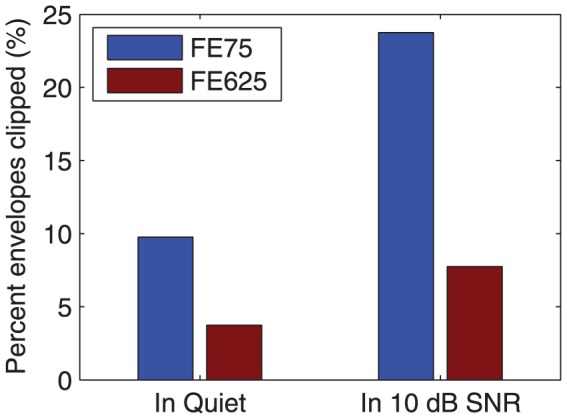
Envelope clipping in experiment 1. Proportion of envelope samples that exceeded the LGF saturation level for sentences presented at 89-talker babble at 10 dB SNR, for the front-end limiter.

Figure 9 shows the proportion of envelope samples that exceeded the LGF saturation level (i.e. the amount of envelope clipping) with the front-end limiter for sentences presented at 89 dB SPL in experiment 1 (results for the envelope profile limiter are not shown because it had zero clipping under all conditions). Much more clipping occurred for sentences in noise than in quiet, because the noise level of 79 dB SPL exceeded the 65 dB SPL compression threshold for speech-like signals. In both quiet and noise, increasing the release time substantially reduced the amount of clipping, because the gain was lower on average.

In experiment 1, when the release time was kept constant, the envelope profile limiter gave speech intelligibility that was at least equivalent to that for the front-end limiter. The speech-in-quiet condition revealed the effect of envelope distortion. With the 75 ms release time, about 10% of envelope samples were clipped (Figure 9), and the envelope profile limiter provided a small benefit (approximately 6 percentage points), perhaps due to better representation of spectral peaks (Figure 5). With the 625 ms release time, clipping affected less than 4% of envelope samples (Figure 9), so there was little scope for the envelope profile limiter to provide benefit. The results suggest that the subjects were not very sensitive to envelope clipping. This is consistent with the results of Khing et al [35], where cochlear implant speech scores at high SNR with no AGC were not significantly degraded until more than 25% of stimulation pulses were affected by envelope clipping. Zeng and Galvin [1] found a relatively small reduction in cochlear implant vowel intelligibility (about 10 percentage points) in noise and in quiet when the electrical dynamic range was reduced to one current level, giving a binary representation, which is equivalent to 100% of the pulses being affected by envelope clipping. It should be noted that these results were obtained with the ACE or SPEAK coding strategies, which select the envelopes with largest amplitude for stimulation in each cycle [5]; it is possible that envelope clipping may be more detrimental in a coding strategy such as Continuous Interleaved Sampling (CIS), which stimulates all channels in each cycle [36].

One methodological issue with experiment 1 was the ceiling effect for sentences in quiet, especially with the 625 ms release time. To better observe a difference between the two AGC types in quiet, more difficult speech material is needed. Isolated words or a vowel confusion test could be used, perhaps with a carrier phrase to exercise the dynamic behaviour of the AGC systems. An alternative is to use low predictability or nonsense sentences [37].

The secondary conclusion of this study is that a short release time (75 ms) led to lower intelligibility than a longer release time (625 ms). The effect was consistent across subjects, and was greatest for speech in noise, with scores in experiment 1 dropping by more than 45 percentage points when the release time was decreased. In experiment 2, it is very likely that the advantage of EP625 over FE75 was primarily due to the longer release time. The consistency and size of the detriment for fast compression with cochlear implants contrasts with the mixed results obtained in studies with acoustic hearing aids [16]. Moore [17] proposed that the benefit of fast compression found for some subjects depended on the individual's ability to process temporal fine structure, which facilitates listening in the dips of background noise. The results of the present study are consistent with that hypothesis, as cochlear implants are unable to convey temporal fine structure.

Release time also had a significant effect for speech in quiet in experiment 1, implying that envelope distortion also played a role. Increasing the release time reduced the amount of envelope clipping (Figure 9). Furthermore, as cochlear implant speech perception relies on envelope cues, the fidelity of the envelopes is important [23]. The modulation frequencies associated with words, syllables and phonemes are around 2.5 Hz, 5 Hz and 12 Hz respectively [38], with envelope modulations in the range 2 – 16 Hz most important for speech intelligibility [39]–[41]. Based on those studies, the AGC release time needs to be at least 500 ms to maintain modulation cues.

Because of the inherent fluctuations in speech and babble, the instantaneous SNR varies with time, and thus time-varying gain has the potential to change the average SNR. In a separate analysis of the results of experiments 1 and 2 [42], an output SNR metric was developed that was a good predictor of sentence scores across the full range of presentation levels. A 75 ms release time degrades the output SNR because lower gain is applied to the high-amplitude speech syllables than to the noise background. With a 625 ms release time, the degradation is not as severe because there is less gain variation over the course of a sentence.

The poor results with the 75 ms release time may explain why Spahr and Dorman [43] found that ESPrit 3G users performed worse in noise than users of the CII or Tempo+ sound processors (which had dual-loop AGC systems). The ESPrit 3G processor (released in 2002), used a front-end compression limiter with a release time of 82 ms, and although ASC was available, it was not enabled in the default processor setting. In contrast, the CP810 processor (released in 2009) has a dual-loop AGC system (the slow stage, ASC, is on by default). The performance-intensity functions of experiment 2 (with ASC disabled) suggest the improvement that would be obtained if ASC was enabled. Based on bench measurements, at 89 dB SPL and 10 dB SNR, ASC would reduce the gain by 18 dB; this is equivalent to reducing the presentation level to 71 dB SPL, and suggests that scores would improve from about 20% correct to 80% correct.

Regarding sound quality, recipients in the take-home study showed no strong preference between the standard (dual-loop) AGC and the (single-loop) envelope profile limiter with 625 ms release time. However, some recipients noticed sound drop-outs following impulsive sounds with the envelope profile limiter. This is a known issue for an AGC with a release time over 300 ms [27], [17]. To alleviate this, the envelope profile limiter could be used in a dual-loop configuration. This requires a slow gain stage to be placed before the envelope profile limiter. Note that in the take-home study, the envelope profile limiter compressed large envelope excursions before ADRO could process them, so the signal path did not operate as a dual-loop system.

To date, cochlear implants have used AGC systems that were essentially the same as those developed for acoustic hearing aids. A short AGC release time appears to have a more detrimental effect in cochlear implants than in hearing aids, showing the importance of studies involving cochlear implant recipients. The envelope profile limiter developed in the present study was specifically tailored to the needs of a cochlear implant system, and would not be suitable for a hearing aid. Moving all the gain control elements to after the filter bank opens new opportunities for optimisation and integration with other processing algorithms.
